# Experimental investigation into mechanical, thermal, and shape memory behavior of thermoresponsive PU/MXene shape memory polymer nanocomposite

**DOI:** 10.1016/j.heliyon.2024.e24014

**Published:** 2024-01-04

**Authors:** Rajita Sanaka, Santosh Kumar Sahu

**Affiliations:** School of Mechanical Engineering, VIT-AP University, Besides A.P. Secretariat, Amaravati 522237, Andhra Pradesh, India

**Keywords:** Shape memory polymer nanocomposite, Polyurethane, MXene, Mechanical properties, Thermal properties, Shape memory behavior

## Abstract

This research presents an experimental investigation into the mechanical, thermal, and shape memory behavior of a thermos-responsive polyurethane (PU) reinforced with 0–1.0 wt % of MXene (Ti_3_C_2_T_x_) nanofiller. The PU/MXene nanocomposites were fabricated using sonication and injection molding route. The 0.5 wt % PU/MXene nanocomposite showed the optimum mechanical properties i.e. tensile modulus, tensile strength, and hardness value, which are improved by 22, 281, and 19 %, respectively, compared to pure PU. The improvement is observed in melting temperature (T_m_), the heat of melting (h_m_), crystallization temperature (T_c_), and the heat of crystallization (h_c_) results. The percentage of crystallinity revealed enhancements of 6 %, 18 %, 24 %, and 34 % for 0.1, 0.2, 0.3, and 0.5 wt % PU/MXene samples respectively compared to pure PU. The findings from the shape recovery experiments demonstrated that the inclusion of MXene has no impact on both the shape fixity and shape recovery performance. The PU/MXene nanocomposite with improved mechanical and thermal properties can find potential applications in robotics actuators, medical devices, sensors, etc.

## 1 Introduction

Shape memory polymers (SMPs) have emerged as a fascinating class of materials due to their unique ability to recover a predefined shape from a temporary one upon exposure to external stimuli. Among various SMPs, thermoresponsive shape memory polymers (SMPs) have gained significant attention owing to their responsiveness to changes in temperature [1]. Polyurethane (PU) is a class of thermoresponsive shape memory polymers widely used owing to their excellent shape memory behavior and tunable properties. However, it has limitations like poor mechanical and thermal properties [2,3], which can be improvised by adding suitable nanofillers in the PU matrix [4,5]. The significant literature that is accessible on this topic is summarised in the section that follows.

The tensile characteristics of the PU nanocomposite were reported by Gohar et al. in Ref. [6], where PU matrix nanocomposite reinforced with 0–1.0 wt % of MWCNT content was used for nanocomposite fabrication. It was observed that there is an increase of 25 % in modulus of elasticity, 21 % in ultimate tensile strength, and 11 % in elongation at break for 1.0 wt % of MWCNT samples compared to pure PU. This was due to the excellent dispersion and interfacial bonding of MWCNTs in the PU matrix. Namathoti et al. [7] demonstrated an improvement in the tensile and thermal properties of PU nanocomposite filled with MWCNT and HNT (0–1 wt %). The highest tensile strength value, i.e., 23.5 MPa, and glass transition temperature of 69 °C is observed for 0.1 wt % PU/MWCNT nanocomposite. The thermal properties of PU/Bentone (B38) clay nanocomposite were evaluated by Babar et al. [8]. The test was performed at three weights % such as 1, 2, and 3 wt% and observed an improvement of 34 % in glass transition temperature and a shift of degradation temperature, i.e., 46 °C at 1 wt % of PU/B38 compared to pure PU. Thiyagu et al. [9] investigated the influence of graphene addition on PU nanocomposite's mechanical, thermal, and shape memory properties. It was concluded that adding graphene improved the tensile modulus, tensile strength, and thermal properties. However, it was discovered that the shape memory capabilities are unaffected by adding graphene. Sharma et al. [10] reported the mechanical properties of PU/C20A clay nanocomposite, where clay content ranges from 0 to 3 wt %. It was observed that, at 3 wt% PU/C20A nanocomposites processed by sonication and homogenization showed a 53 % decrease in Young's modulus but increased elongation at the break by 97 % and toughness by 93 % compared to pure PU. The thermal properties of chemically modified CNT (0.01–2 wt%) in the PU matrix were investigated by Haji et al. [11]. It was noted that the surface-modified CNT improvised the mechanical and thermal properties of PU/CNT nanocomposite. Mohammadzadeh et al. [12] investigated the role of halloysite nanotubes (HNT) on the shape recovery behavior of PU nanocomposite by varying 1 to 2 wt % of HNT. It was examined that the addition of 1 wt% HNT the shape fixity ratio was raised due to soft phase purity, while the shape recovery parameter was decreased due to reduced chain mobility. Albozahid et al. [13] showed the results of adding GNPs (0.25–0.75 wt%) to the PU matrix, Young's modulus was seen to an increase of 50 %, 81 %, and 127 % with 0.25, 0.5, and 0.75 wt % of GNP, respectively. Superior dispersion and interfacial GNP adhesion to the PU matrix were cited as the reasons for this. A TPU matrix reinforced with MXene (Ti_3_C_2_T_x_) loading at 0–1 wt% was studied by Gao et al. [14]. It was demonstrated that a 0.5 wt% MXene loading increases tensile strength and elongation at the break by 15.4 and 41.2 %, respectively.

The above section concludes that most of the research focused on polyurethane matrix reinforced with graphene, MWCNT, HNT, GNP, etc., with a small number on MXene nanofiller. The objective of the investigation is to comprehensively investigate the mechanical, thermal, and shape recovery behavior of PU/MXene thermoresponsive shape memory polymer nanocomposites. The choice of MXene (Ti_3_C_2_T_x_) is owing to its remarkable mechanical properties, which possess Young's modulus of about 330 GPa [15], highlighting the exceptional stiffness and strength that MXene can impart to the PU composite. The MXene exhibits high thermal conductivity [16], significantly higher than pure PU. This quantitative difference in thermal conductivity demonstrates the potential for MXene to enhance the composite's ability to efficiently conduct and dissipate heat, which is crucial for thermal response [15,16]. The deliberate integration of MXene underscores the research's novelty as nanofillers into the PU matrix, a distinctive approach that imparts unparalleled properties and functionalities to the nanocomposites. This novel integration, substantiated through the extensive investigation encompassing mechanical, thermal, and shape recovery tests, contributes to a notable performance enhancement over conventional shape memory polymers.

## 2 Materials and methods

### 2.1 Materials

SMP Technologies from Japan supplied the polyurethane (PU) required. Nano Research Elements from India is the supplier of MXene (Ti_3_C_2_T_x_). Table 1 displays detailed technical data about PU and MXene.

**Table 1:** Materials specifications as supplied.

Materials	Technical Specification
Polyurethane (PU)	Pallet size: 6–8 mm; Glass transition temperature (T_g_): 55 °C, Density 0.83 g/cm^3^;
MXene	Particle size: 40–50 nm, Form: flakes, Purity>99 %, Density: 3.8 g/cm^3^

### 2.2 Scanning electron microscopy (SEM)

**Fig. 1:**
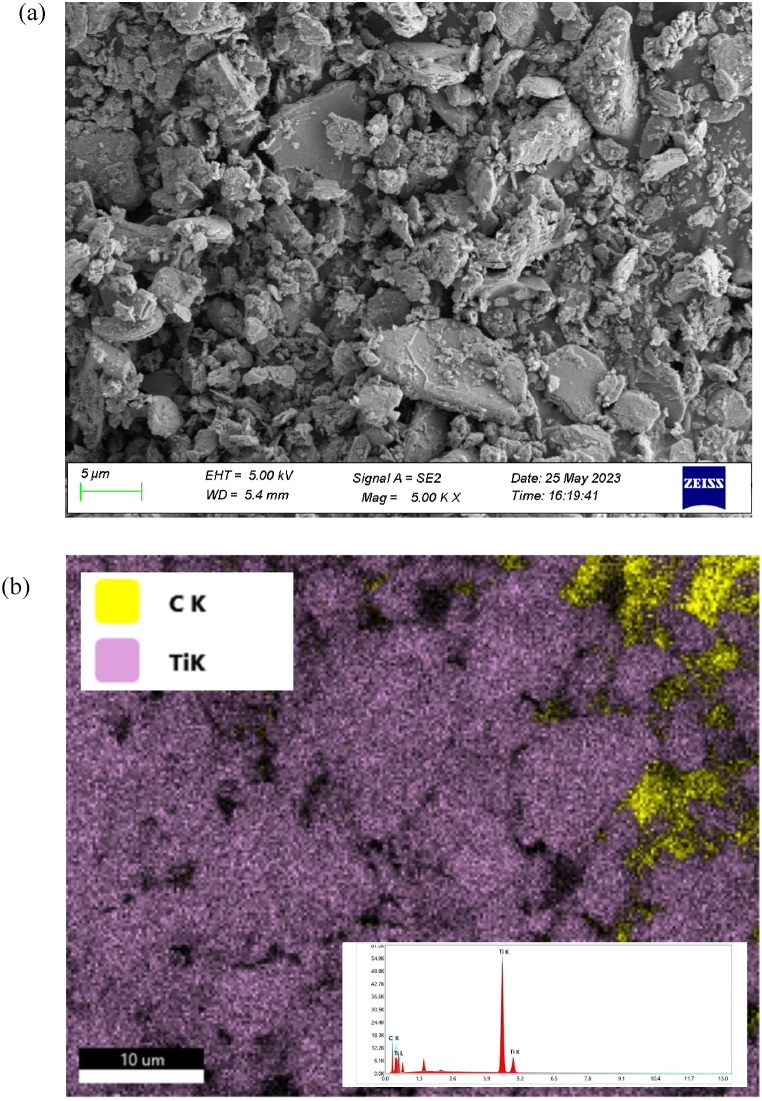
a) SEM image of MXene nanopowder; b) EDX of MXene nanopowder.

Scanning electron microscopy (SEM) and Energy dispersive X-ray spectroscopy (EDX) tests were conducted using Carl Zeiss Supra 55 equipment. The SEM test is performed on MXene (Ti_3_C_2_T_x_) nano-powder, which reveals the flake structure of MXene as shown in Fig. 1a. The elemental composition of MXene nanomaterial is ascertained by the presence of Titanium (Ti) and carbon (C) through EDX test as shown in Fig. 1b.

### 2.3 Fabrication of composite sample

**Fig. 2:**
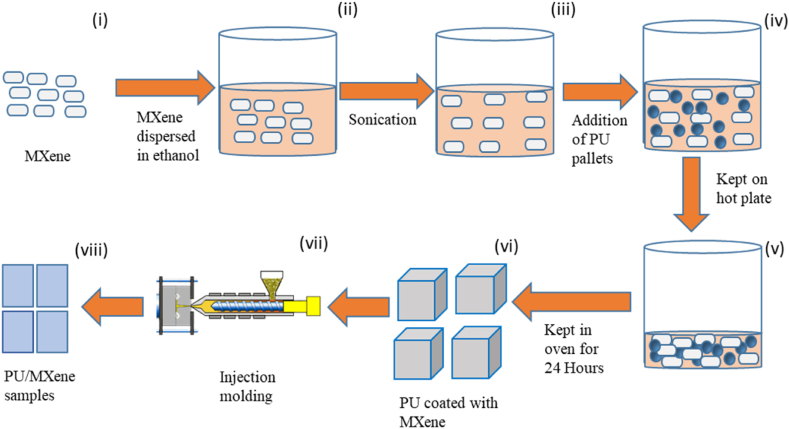
Fabrication route implemented.

The composite samples were polyurethane (PU) reinforced with 0.1, 0.2, 0.3, 0.5, and 1.0 wt % of MXene. Fig. 2 depicts the manufacturing procedure implemented to fabricate PU/MXene nanocomposite. Firstly, the requisite weight % of MXene is taken in a beaker and is chemically modified, as described in the literature by Cao et al. [17]. Then a beaker filled with the ethanol base and the appropriate weight fraction of chemically modified MXene nanoplatelets was added to the solution at 10:1. The mixture is then agitated while being held over a sonicator to ensure a uniform dispersion solution. Subsequently, the nano-solution obtained from the sonication process was added to the PU pallets, and thorough mixing was performed on a hot plate to promote homogeneity. The sample was kept in an oven for 24 h to allow for complete evaporation of the ethanol bases, thus minimizing any potential moisture stress. The PU coated with MXene nanofillers thus obtained was fed through a hopper of a twin screw injection-molding machine.

Finally, the samples are extracted as rectangular sheets from the injection molding machine. The fabricated samples are denoted as 0.1 PU/MXene, 0.2 PU/MXene, 0.3 PU/MXene, 0.5 PU/MXene, and 1.0 PU/MXene.

### 2.4 Mechanical test

The tensile tests were performed using UTM testing equipment (Tinius Olsen H10 KL) following ASTM D638. All samples were evaluated at room temperature, and a 2 mm/min crosshead movement was used. Each test sample underwent five rounds of testing, and the average result was recorded. The micro-hardness investigations were performed through Vickers Diamond Pyramid Indenter HV-1000Z. A load of 1 kg over a dwell period of 20 s is maintained throughout the test. There were a total of five indentations made to ascertain the repeatability.

### 2.5 Thermal test

A simultaneous thermal analyzer (SAT 8000 from PerkinElmer) with a temperature range of 25–600 °C and a heating and cooling rate maintained at 20 C/min was used to perform the differential calorimetry (DSC) and thermogravimetric (TGA) study. It was utilized with a sample with a weight of around 25 mg. A nitrogen gas environment is used for the test, and a constant flow rate of 20 mL/min of nitrogen gas is used. There were a total of five repetitions of tests performed for each sample to ascertain the repeatability.

### 2.6 Thermoresponsive shape memory test

The study investigates the thermoresponsive shape memory characteristics using the fold-deploy method on rectangular specimens (80 × 10 × 2 mm) [18]. The experimental process involved several steps: (i) the samples were heated above the glass transition temperature (T_g_), i.e., 80 °C on a heater for 5 min (ii) Afterward, the samples were carefully shaped into a "U" form and held in place temporarily. (iii) To maintain the external force, the "U"-shaped samples were quickly submerged in a water bath at room temperature. The initial angle of deformation (θ_i_) was then noted. (iv) The samples were then placed in a water bath at a temperature higher than Tg, i.e., 80 °C, and the shape change with time (t) was monitored and the final angle of recovery (θ_f_) was also recorded. The test is repeated for three cycles. In the evaluation of the safe fixity rate (R_f_), and shape recovery rate (R_r_), the relevant equations (1), (2)) were utilized [19]:

(1) $$R_{f}=\frac{\left(\theta_{i}-\theta_{f}\right)}{\theta_{i}}\times 100\%$$

(2) $$R_{r}=\frac{\theta_{f}}{180{}^{\circ}}\times 100\%$$

The symbols used above carry the same meaning as in literature [19].

## 3 Results and discussion

### 3.1 Mechanical properties

**Fig. 3:**
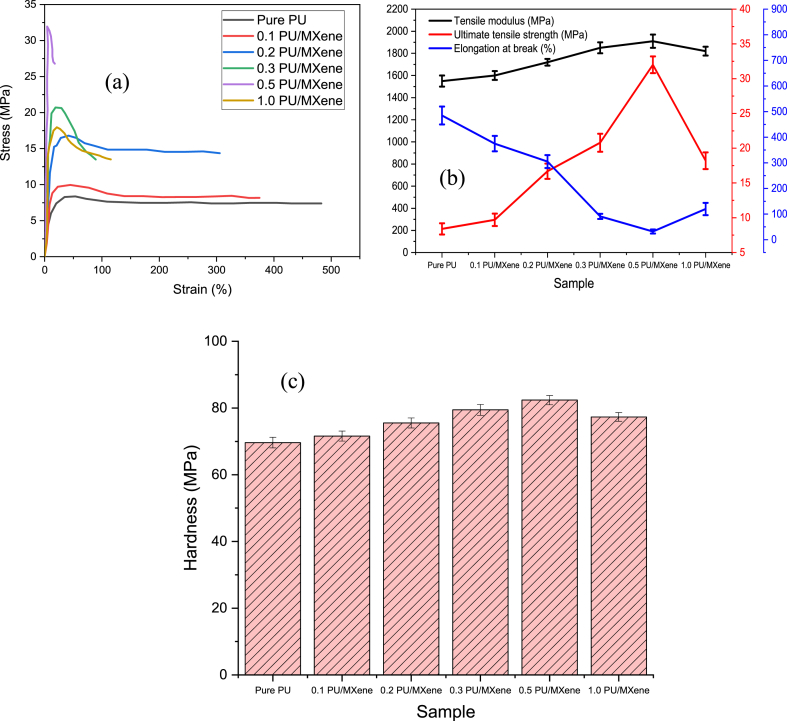
a) Stress vs. strain; b) Tensile properties; c) Hardness results for all the sample.

The stress-strain curve depicts the relationship between the applied stress and the material's resultant strain. It offers insightful information about the performance and mechanical behavior of the nanocomposite. From Fig. 3a, it is clear that all the nanocomposite samples approach linear deformation, and further loading causes them to reach an ultimate point and final breaking point. The 0.5 wt% PU/MXene nanocomposite demonstrates the steepest slope, demonstrating the high load necessary to generate deformation compared to all the other samples. The mechanical properties such as tensile modulus, ultimate tensile strength, and % elongation at break were obtained from the stress-strain curve and represented in Fig. 3b. The lowest tensile modulus is observed for pure PU, which is increased further by 3, 11, 19, and 22 % for 0.1 PU/MXene, 0.2 PU/MXene, 0.3 PU/MXene, and 0.5 wt% PU/MXene respectively. A similar trend is observed in the ultimate tensile strength results as well, which shows an increase of 15, 99, 147, and 281 % for 0.1, 0.2, 0.3, and 0.5 wt % of PU/MXene in comparison with pure PU, respectively.

**Fig. 4:**
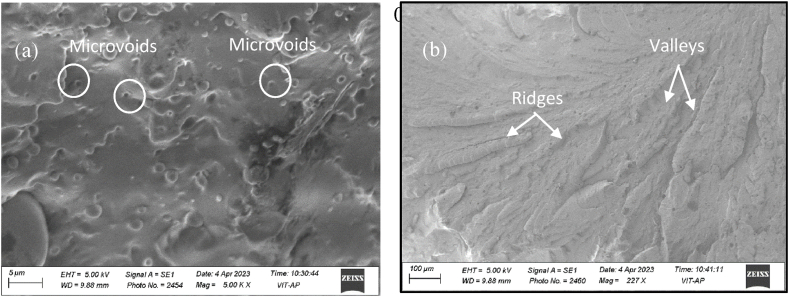
Fractography image of a) Pure PU; b) 0.5 wt% PU/MXene.

However, for 1.0 wt% of PU/MXene, there is a decrease in tensile modulus and tensile strength is noted compare to 0.5 wt% of PU/MXene. The increase in tensile strength with an increase in MXene concentration up to 0.5 wt % is attributed to the uniform dispersion of MXene nanofillers in the PU matrix along with good interfacial adhesion between the matrix and the nanofiller [20]. However, the further increase in MXene (i.e. beyond 0.5 wt %) of filler in PU resulted in agglomeration and hence caused a decrease in tensile modulus and tensile strength [21]. The highest value of % elongation is seen for pure PU and follows the trend Pure PU > 0.1 PU/MXene> 0.2 PU/MXene>1.0 PU/MXene >0.3 PU/MXene>0.5 wt % PU/MXene. The reason for a higher value of % elongation for pure PU is due to its flexible and elastomeric nature. In addition, it has a molecular structure that allows for significant chain mobility, enabling the material to undergo large deformations [22]. This large deformation allows pure PU to exhibit high elongation percentages before failure. Fig. 3c shows the hardness result for all the samples, which is performed to evaluate the influence of MXene in PU. The lowest hardness value is observed for PU, i.e., 69 MPa, which is increased by 3, 9, 14, 19, and 11 % for 0.1, 0.2, 0.3, 0.5, and 1.0 wt % of MXene. respectively. The reason for the highest value of hardness for 0.5 wt % PU/MXene is related to the % crystallinity value, explained in the subsequent section. The 0.5 PU/MXene nanocomposite exhibited the optimum mechanical properties, hence up to 0.5 wt % MXene in PU is adopted to evaluate fractography, thermal properties, and shape memory behavior. The fractography study is performed to understand the failure mechanisms for pure PU and 0.5 wt% PU/MXene sample after the tensile test through a scanning electron microscope (SEM), as shown in Fig. 4. It is observed that for Pure PU as shown in Fig. 4a, the fracture is ductile in nature, which is characterized by microvoids [23]. The microvoids undergo progressive filling up with the increase in MXene content. And at 0.5 wt% MXene content as shown in Fig. 4b, the fractography study indicated that the nanocomposite turns brittle, characterized by river pattern, where the fracture surface exhibits a series of ridges and valleys.

### 3.2 Thermal properties

**Fig:5:**
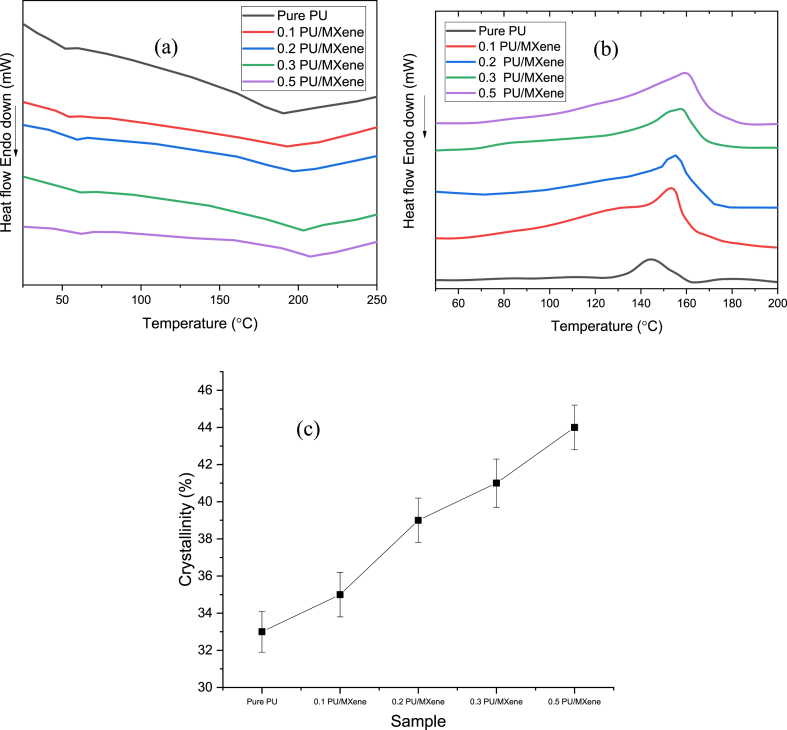
a) Heating curve; b) Cooling curve; c) % Crystallinity for all the sample.

Differential scanning calorimetry (DSC) is performed to evaluate the thermal transition and crystallinity properties of pure PU and PU/MXene nanocomposite. The thermal transition is analyzed through a heating and cooling curve. Fig. 5a shows the heating curve, which is used to extract the melting temperature (i.e., T_m_) and heat of melting (h_m_) for all the samples, as shown in Table 2. It is observed that for pure PU, the T_m_ is 191 °C, which is increased to 193, 197, 203, and 207 °C for 0.1 PU/MXene, 0.2 PU/MXene, 0.3 PU/MXene, and 0.5 wt% PU/MXene, respectively. A similar trend is observed in the h_m_ results as well. This is because adding MXene nanofillers can influence the molecular arrangement of the PU chains, promoting chain alignment and ordering [24]. A more ordered molecular structure in the PU contributes to a higher T_m_ and h_m_ value, as more energy is required to break the ordered arrangements during the melting process. Similarly, Fig. 5b shows the cooling curve, which is used to extract the crystallization temperature (T_c_) and heat of crystallization (h_c_) and is represented in Table .2. The lowest T_c_ is observed for pure PU and follows the trend Pure PU < 0.1 PU/MXene< 0.2 PU/MXene<0.3 PU/MXene<0.5 wt % PU/MXene. A similar trend is observed in h_c_ results as well. The higher value of T_c_ and h_c_ is reasoned due to its high surface area and unique interlayer spacing, providing favorable nucleation sites and promoting ordered polymer packing during crystallization [25]. The % crystallinity computed per equation (3) for all the samples and is shown in Fig. 5c and Table 2 [26].

(3) $$\chi_{c}\left(\%\right)=\frac{\Delta h_{c}}{\Delta h_{c}^{0}}$$

Where $\Delta h_{c}$ and $\Delta h_{c}^{0}$ are the heat of crystallization (J/g) of the test sample and heat of crystallization (J/g) of 100 % crystalline polymer sample, respectively.

**Table 2:** Parameters obtained from the DSC test.

Samples	T_m_ (ͦC)	T_c_ (ͦC)	h_m_(J.g^−1^)	h_c_(J.g^−1^)	χ_c_ (%)
Pure PU	191	144	120	56	33
0.1 PU/MXene	193	145	131	62	35
0.2 PU/MXene	197	147	145	68	39
0.3 PU/MXene	203	150	152	71	41
0.5 PU/MXene	207	152	165	78	44

The % crystallinity for pure PU is 33 % which increased by 6, 18, 24, and 34 % for 0.1 PU/MXene, 0.2 PU/MXene, 0.3 PU/MXene, and 0.5 wt% PU/MXene, respectively. This is owing to the presence of MXene nanoparticles, which may disrupt the chain entanglements in the polyurethane matrix. Reduced entanglements facilitate ordered crystalline regions forming during cooling, contributing to a higher % crystallinity [27].

**Fig. 6:**
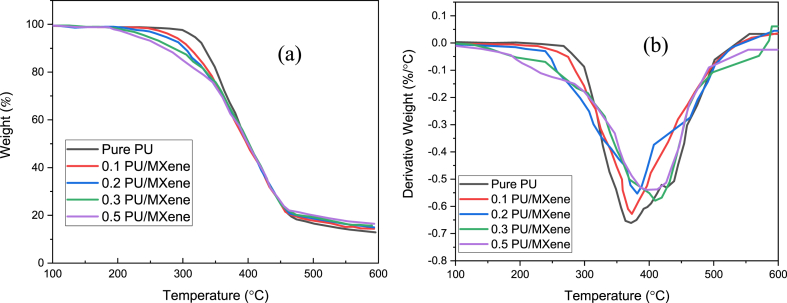
a) TGA curve; b) DTG curve for all sample.

The thermal decomposition for all the samples is analyzed through the TGA curve, as shown in Fig. 6a. It is observed that the highest value of onset temperature is seen for 0.5 wt% PU/MXene, i.e., 247 °C, which is decreased to 231, 206, 193, and 185 °C for 0.3 PU/MXene, 0.2 PU/MXene, 0.1 PU/MXene, and pure PU respectively. The reason for this owing to the presence of MXene, may create a protective barrier, hindering the diffusion of volatile species and delaying the onset of decomposition, thus leading to a rise in onset temperature for 0.5 wt% PU/MXene nanocomposite [28]. The DTG curve is the derivative of the TGA curve. It represents the rate of change of weight (mass) concerning temperature, as shown in Fig. 6b. The degradation temperature for all samples is obtained, corresponding to the peak in the DTG curve. The highest value of degradation temperature is observed for 0.5 wt% PU/MXene, i.e..

413 °C, decreased to 410, 382, 373, and 370 °C for 0.2 PU/MXene, 0.1 PU/MXene, 0.05 PU/MXene, and pure PU, respectively. This is due to the interfacial interaction between MXene nanoparticles and the PU matrix, which can enhance degradation temperature [29].

### 3.3 Shape memory behavior

**Fig. 7:**
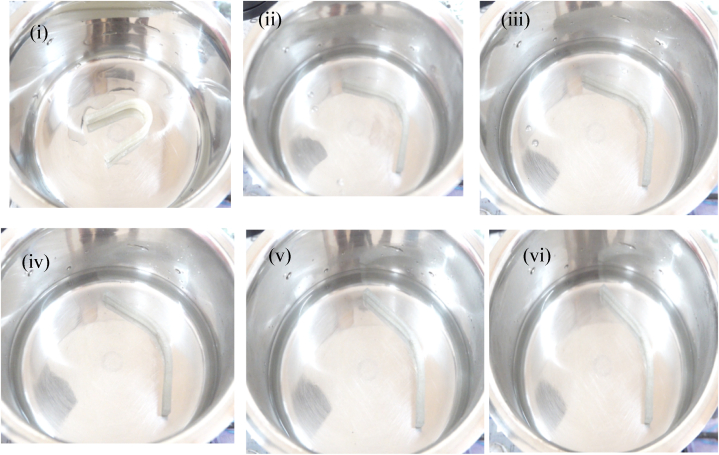
Shape memory test for Pure PU at various time frames of i) 10, ii) 20, iii) 30, iv) 40, v) 50, and vi) 60 s at cycle 1.

**Fig. 8:**
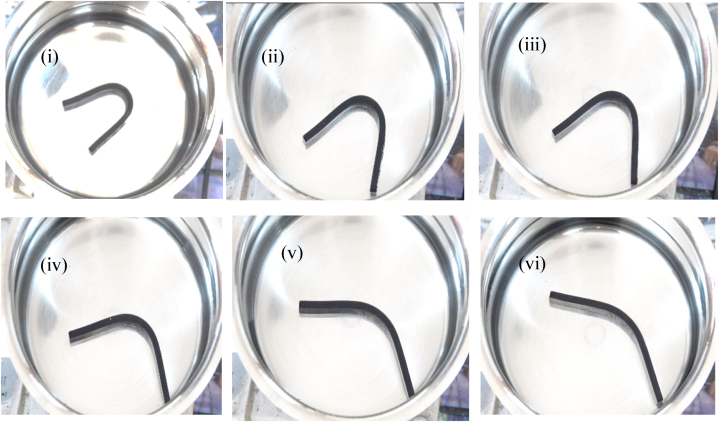
Shape memory test for 0.5 wt % PU/MXene at various time frames of i) 10, ii) 20, iii) 30, iv) 40, v) 50, and vi) 60 s at cycle 1.

**Fig. 9:**
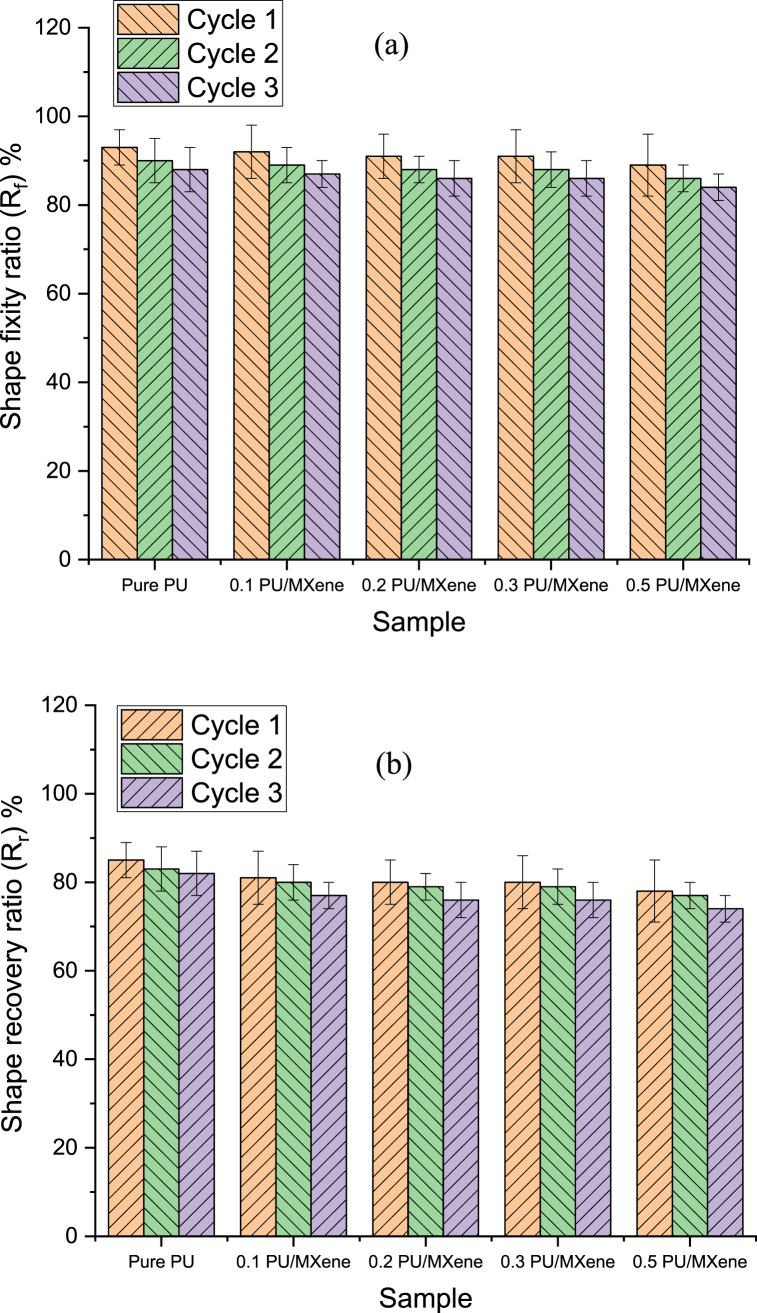
a) Shape fixity ratio; b) Shape recovery ratio for all the sample.

The shape recovery test evaluates the material's memorization ability to achieve the permanent shape in response to three cycles at 80 °C test conditions. Fig. 7, Fig. 8 depict the sample deformation shape of Pure PU and 0.5 wt% PU/MXene at various time frames of 10, 20, 30, 40, 50, and 60 s at cycle 1. The results are extracted from the deformation states as per section 2.6 explained before and represented in Fig. 9a–b, Fig. 9a shows that at cycle 1 test condition, the shape fixity ratio for pure PU is 88 %, which is decreased by 1, 2, 3, and 4 % for 0.1 PU/MXene, 0.2 PU/MXene, 0.3 PU/MXene, and 0.5 PU/MXene respectively. A similar trend is observed in cycle 2 and cycle 3 as well. The shape recovery results represented in Fig. 9b, show that at cycle 1 for pure PU is 82 %, which is decreased by 2, 3, 4, and 5 % for 0.1 PU/MXene, 0.2 PU/MXene, 0.3 PU/MXene, and 0.5 wt % PU/MXene, respectively. A similar trend in shape recovery results are observed at cycle 2 and cycle 3 conditions as well. The.

reason for the decrease in shape fixity and shape recovery ratio for PU/MXene composite owing to the hindering of chain mobility of the polyurethane matrix with the presence of MXene, and the higher concentration of MXene tends to decrease the chain mobility further [30].

## 4 Conclusion

The research is on the experimental investigation into the mechanical, thermal, and shape memory behavior of a thermoresponsive polyurethane (PU) nanocomposite reinforced with 0–1.0 wt % MXene (Ti_3_C_2_T_x_) content. The following conclusion is drawn from this research-

i. Adding MXene (Ti_3_C_2_T_x_) content to polyurethane (PU) resulted in significant improvements in mechanical properties, up to 0.5 wt % PU/MXene nanocomposite.
ii. Thermal properties for 0.5 wt % PU/MXene were enhanced, with improvements observed in melting temperature (T_m_), the heat of melting (h_m_), crystallization temperature (T_c_), and the heat of crystallization (h_c_).
iii. The % crystallinity also increased for 0.5 wt % PU/MXene, indicating improved structural ordering in the nanocomposite.
iv. No improvement in shape memory behavior is observed by incorporating MXene in PU matrix.

## Data availability statement

No data was used for the research described in the article.

## CRediT authorship contribution statement

**Rajita Sanaka:** Writing – original draft, Visualization, Methodology, Investigation, Formal analysis, Conceptualization. **Santosh Kumar Sahu:** Writing – review & editing, Supervision, Methodology, Funding acquisition, Formal analysis, Conceptualization.

## Declaration of competing interest

None.
